# The role of cardiolipin in mitochondrial dynamics and quality control in neuronal ischemia/reperfusion injury

**DOI:** 10.1038/s41419-025-07786-8

**Published:** 2025-07-05

**Authors:** Katlynn J. Emaus, Garrett M. Fogo, Joseph M. Wider, Thomas H. Sanderson

**Affiliations:** 1 Neuroscience Graduate Program, University of Michigan, Ann Arbor, MI USA; 2 Department of Emergency Medicine, University of Michigan, Ann Arbor, MI USA; 3 Department of Neurology, Brigham and Women’s Hospital, Boston, MA USA; 4 The Max Harry Weil Institute for Critical Care Research and Innovation, University of Michigan, Ann Arbor, MI USA; 5 Department of Molecular and Integrative Physiology, University of Michigan, Ann Arbor, MI USA

**Keywords:** Molecular neuroscience, Cardiovascular diseases

## Abstract

Stroke and cardiac arrest claim the lives of millions worldwide each year emphasizing the importance of understanding this injury cascade. These pathologies present as a ‘two hit’ injury termed ischemia/reperfusion (I/R) injury. The primary injury is the initial disruption of blood flow and ischemic state while the secondary injury, paradoxically, being the return of blood flow and oxygen availability. The injury caused by reperfusion presents a viable window for therapeutic intervention, stressing the importance of understanding this injury pathology. Constantly undergoing fission and fusion, mitochondria are dynamic organelles that play a vital role in maintaining cell health and are highly susceptible to I/R injury. Following I/R injury, disrupted mitochondrial dynamics and quality control ultimately lead to a dysfunctional mitochondrial network, energy depletion and eventually cell death. While mitochondrial dynamics and quality control have been studied extensively in the realm of I/R injuries, the role of mitochondrial lipids is emerging as an important component of injury progression. The inner mitochondrial membrane lipid, cardiolipin has been demonstrated to play an integral role in maintaining mitochondrial quality control, dynamics and energy production. In response to oxidative stress, cardiolipin has been shown to interact with several important proteins involved in mitochondrial dynamics while also contributing to integral signaling cascades. This review will highlight the role of cardiolipin in mitochondrial dynamics and quality control in response to neuronal I/R injury.

## Facts

- Mitochondria are dynamic organelles that constantly undergo fission and fusion to maintain a healthy mitochondrial network and cellular homeostasis.
- Following ischemia/reperfusion injury, mitochondrial dynamics are impacted resulting in a dysfunctional mitochondrial network, inadequate energy reserves and impaired cell health.
- Mitophagy, the selective degradation of damaged mitochondria can be either neuroprotective or detrimental during reperfusion, demonstrating the fragility of the mitochondrial network.
- Cardiolipin, an essential phospholipid found almost exclusively on the inner mitochondrial membrane, contributes to mitophagy and mitochondrial dynamics following neuronal ischemia/reperfusion injury.
- Through its interaction with key dynamic proteins and its role in mitophagy, cardiolipin is through to contribute to neuronal ischemia/reperfusion injury pathology.

## Introduction

Cerebral ischemia/reperfusion (I/R) injuries, including stroke and cardiac arrest, are leading causes of death and disability worldwide [1, 2]. The mammalian brain requires oxygen, glucose, and other metabolites supplied through cerebral circulation for metabolism and function [3–5]. During ischemia, blood flow to the brain is disrupted, leading to depletion of ATP and an eventual state of energy deprivation. Cerebral ischemia induces primary injury via neuronal damage, glial activation, and endothelial cell dysfunction [6–8]. Prompt reperfusion to restore oxygen and nutrient delivery is critical for limiting ischemic injury [9–11]. Reperfusion is necessary to salvage ischemic tissue, however it also induces secondary cellular death and delayed cerebral injury [12]. Reperfusion injury is a significant contributor to brain damage related to cerebral ischemia, however progressive development of the injury offers a treatment window to improve outcomes. Understanding the mechanisms driving I/R injury is critical to advancing our ability to limit reperfusion injury.

The energy production capacity and overall health of a cell are dependent on the maintenance of mitochondrial homeostasis. The mitochondrial life cycle is dictated by several physiological mechanisms, including both mitochondrial dynamics and quality control. Mitochondria are dynamic agents that constantly undergo fission and fusion to maintain mitochondrial architecture [13]. Fission acts to segment mitochondria for the isolation and removal of damaged proteins, mitochondrial DNA mutations, and dysfunctional components [14, 15]. In contrast, fusion constructs and stabilizes mitochondrial networks to promote efficient energy production and mitochondrial matrix equilibrium [16, 17]. Together, fission and fusion dynamics coordinate the mitochondrial network to protect and preserve efficient function and homeostasis [18]. Mitochondrial quality control acts to maintain healthy mitochondria while selectively degrading dysfunctional components through mitophagy. Under ischemic stress, mitochondria release damage associated with molecular patterns (DAMPs) (ie mDNA, TNFa, IL-6) that trigger inflammatory pathways which activate microglia and astrocytes further contributing to the immune response. Following I/R injury, the electron transport chain (ETC) produces mitochondrial reactive oxygen species (ROS). This initiates several intracellular cascades, including an increase in intracellular calcium which can cause opening of the mitochondrial permeability transition pore (mPTP), which contributes to the collapse of the membrane potential. These mechanisms play a critical role in disrupting mitochondrial dynamics and quality control, resulting in a dysfunctional mitochondrial network and inadequate energy reserves.

Recently, lipids have been shown to play a crucial role in mitochondrial quality control and mitophagy [19–23]. Much like proteins, lipids undergo peroxidation when exposed to ROS. Peroxidation alters the physical structure and composition of lipids which can disrupt the structural integrity of cellular membranes [24]. As a double membrane organelle prone to ROS generation, the mitochondria is highly susceptible to structural disruption of either the inner mitochondrial membrane (IMM) or the outer mitochondrial membrane (OMM). Cardiolipin (CL) is an essential phospholipid found almost exclusively in the inner membrane of the mitochondria contributing to cristae structure and cellular respiration. In addition to maintaining membrane integrity, cardiolipin plays mechanistic roles in mitochondrial dynamics, quality control, programmed cell death and cellular respiration [22]. The aim of this review is to detail how CL contributes to the interactions between mitochondrial dynamics and quality control under basal conditions and in response to neuronal I/R injury.

### Cardiolipin metabolism and function

CL is composed of a glycerol head group and two phosphatidate moieties consisting of four alkyl chains. This allows for two forms of asymmetry: molecular and trans-membrane asymmetry. The molecular asymmetry makes CL structurally complex with a variety of subspecies that express tissue specificity. Due to the two chiral centers, two CL subspecies that have four, molecularly identical acyl chains will still maintain two chemically distinct phosphatidyl moieties [25]. The trans membrane asymmetry refers to the uneven distribution of CL between the IMM and the OMM. CL is found almost exclusively in the IMM where it plays a crucial role in supporting membrane curvature. Due to this dimeric structure, cardiolipin holds a conical shape that when clustered, this shape supports cristae curvature and plays an important role in inner membrane support. The overall energy demand of the membrane decreases when lipids of high intrinsic curvature, such as CL, form clusters at polar regions of the membrane, stabilizing the geometric curve of the IMM [26]. Within the inner membrane, CL has been shown to bind and stabilize complex III and IV within the ETC [22, 27]. When bound CLs are disrupted, the ETC becomes inactive and the ETC subunits dissociate [28]. The proposed mechanism by which CL regulates programmed cell death and autophagy is through its affinity for cytochrome c. Under basal conditions, cytochrome c is tethered to CL in the IMM, but when exposed to oxidative stress, CL is externalized, facilitating release of cytochrome c into the cytosol, and inducing programmed cell death [29, 30].

**Fig. 1:**
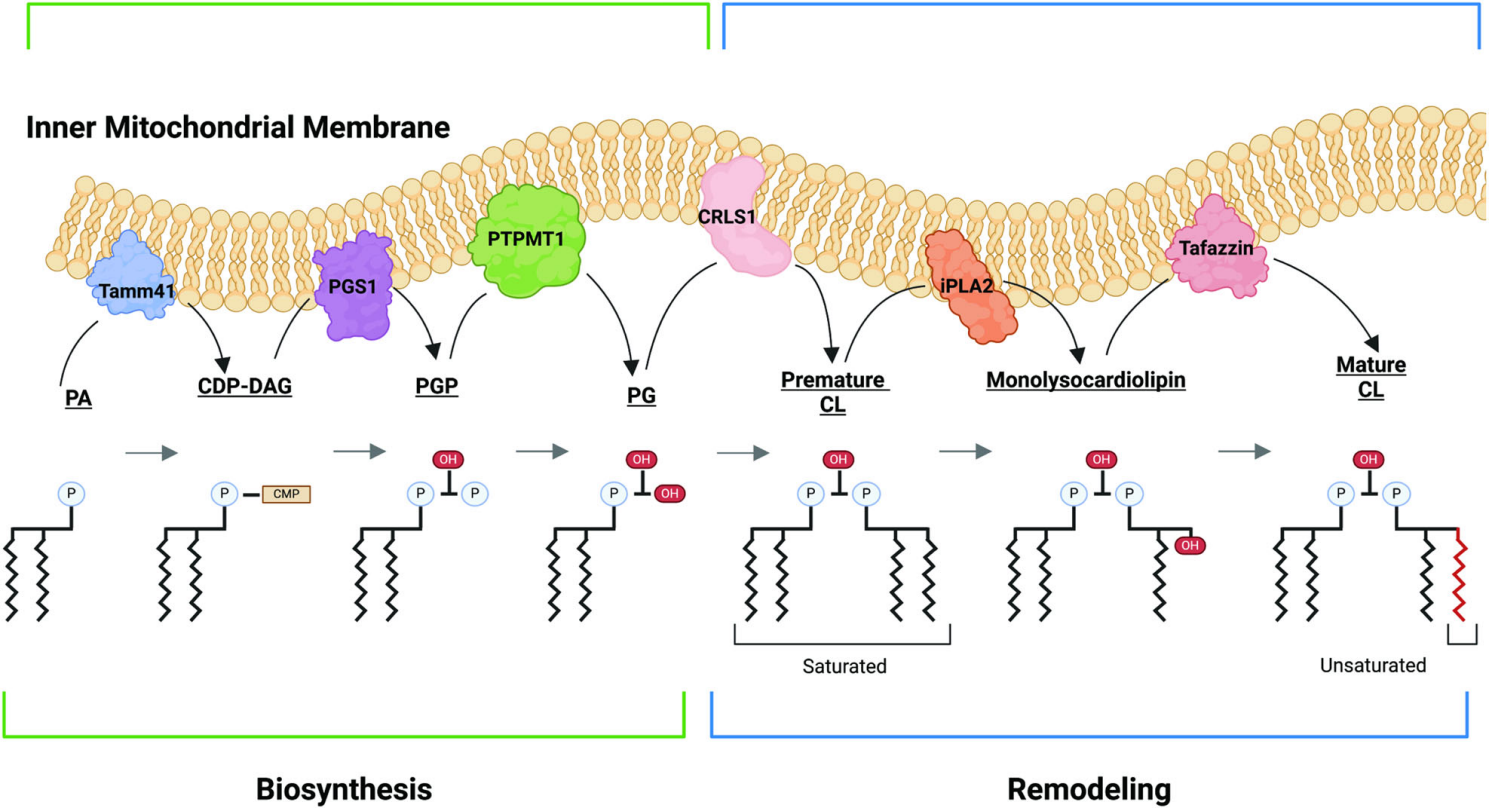
After phosphatidic acid (PA) is imported into the matrix, the cardiolipin biosynthesis process begins. Biosynthesis begins with PA being converted to cytidine diphosphate diacylglycerol (CDP-DAG) by the membrane bound protein Tamm41. CDP-DAG then interacts with phosphatidyl glycerophosphate synthase 1 (PGS1), another membrane bound protein, producing p-glycoprotein (PGP). Protein tyrosine phosphatase mitochondrial 1 (PTPMT1) then converts PGP to PG. Biosynthesis is then concluded with cardiolipin synthase (CRLS1) converts PG to premature cardiolipin (CL). Once premature cardiolipin is produced the remodeling process begins. A membrane of the iPLA_2_ family cleaves one of the fatty acyl chains of the premature cardiolipin and replaces it with a hydroxyl group producing the intermediate monolysocardiolipin (MLCL). Tafazzin then re-acylates monolysocardiolipin with an unsaturated fatty acid chain producing the more stable mature CL. The main difference between premature and mature cardiolipin is the degree of saturation, with the mature form consisting of more unsaturated fatty acid tails resulting in a more stable cardiolipin.

The biosynthesis of CL begins with phosphatidic acid (PA) import into the mitochondrial matrix and conversion to cytidine diphosphate diacylglycerol (CDP-DAG) by the membrane bound protein Tamm41. Phosphatidyl glycerophosphate synthase 1 (PGS1) converts CDP-DAG into p-glycoprotein (PGP) which is then converted to phosphatidylglycerol by protein tyrosine phosphatase mitochondrial 1 (PTPMT1). PG is then converted to premature CL by cardiolipin synthase 1 (CRSL1). The production of premature CL signals the end of the “biosynthesis” phase and marks the “remodeling” phase (Fig. 1). At the conclusion of biosynthesis, premature CL is formed and then remodeled into mature CL. The distinguishing feature between premature and mature CL is the degree of saturation of the fatty acid tails [31]. Following biosynthesis, the structural remodeling process cleaves and re-acylates the fatty acids of CL. Remodeling is driven by the enzyme Tafazzin (TAZ), a transacylase that transfers fatty acids from phospholipids to lysophospholipids [32]. During remodeling, saturated fatty acid tails of premature CL are cleaved by a member of the iPLA_2_ family to form the intermediate monolysocardiolipin (MLCL). TAZ re-acylates MLCL with unsaturated fatty acid tails to form mature CL (Fig. 1). When this remodeling process is disrupted, MLCL accumulation can contribute to mitochondrial dysfunction through lipid pore formation and collapse of the membrane potential [33]. The accumulation of MLCL through TAZ dysfunction is extensively studied in cardiac tissue due to its relevance in Barth Syndrome and contribution to fatal dilated cardiomyopathy [34]. However, its role in neuronal I/R injury is largely unknown. In one study, juvenile rats exposed to global cerebral I/R injury resulted in an increase of caspase 3/7 activation and hydrolysis of CL to lyso- and MLCL [35]. The accumulation of MLCL is thought to dysregulate mitophagy, contributing to dysfunctional mitochondrial dynamics and quality control by disrupting cristae curvature and membrane integrity [36].

### Mitochondrial dynamics

**Fig. 2:**
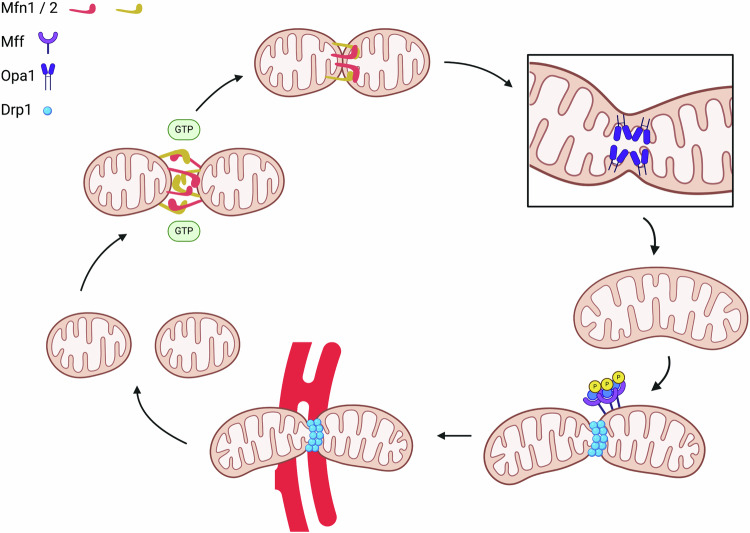
Mitochondrial fusion (upper row) begins with GTP-activated unfolding of Mfn1/2 monomers on adjacent mitochondria. Dimerization of mitofusin 1 and 2 (Mfn1/2) brings two mitochondria closer together to begin outer membrane fusion. Inner mitochondrial membrane fusion is driven by optic atrophy 1 (Opa1), which creates protrusions on the inner membrane and begin matrix fusing. Mitochondrial fission (lower row) is actuated by post-translational modification of dynamin-related protein 1 (Drp1) and accumulation of Drp1 receptors on the outer mitochondrial membrane. Drp1 forms an oligomeric ring around the site of fission. ER tubules come in close association with the fission site to complete division.

As dynamic organelles, mitochondrial structure is continually regulated by several mechanisms to optimize energy production for the cell. Mitochondrial dynamics ensure that the network responds to the needs of the cell through fission and fusion (Fig. 2).

#### Mitochondrial fission

**Fig. 3:**
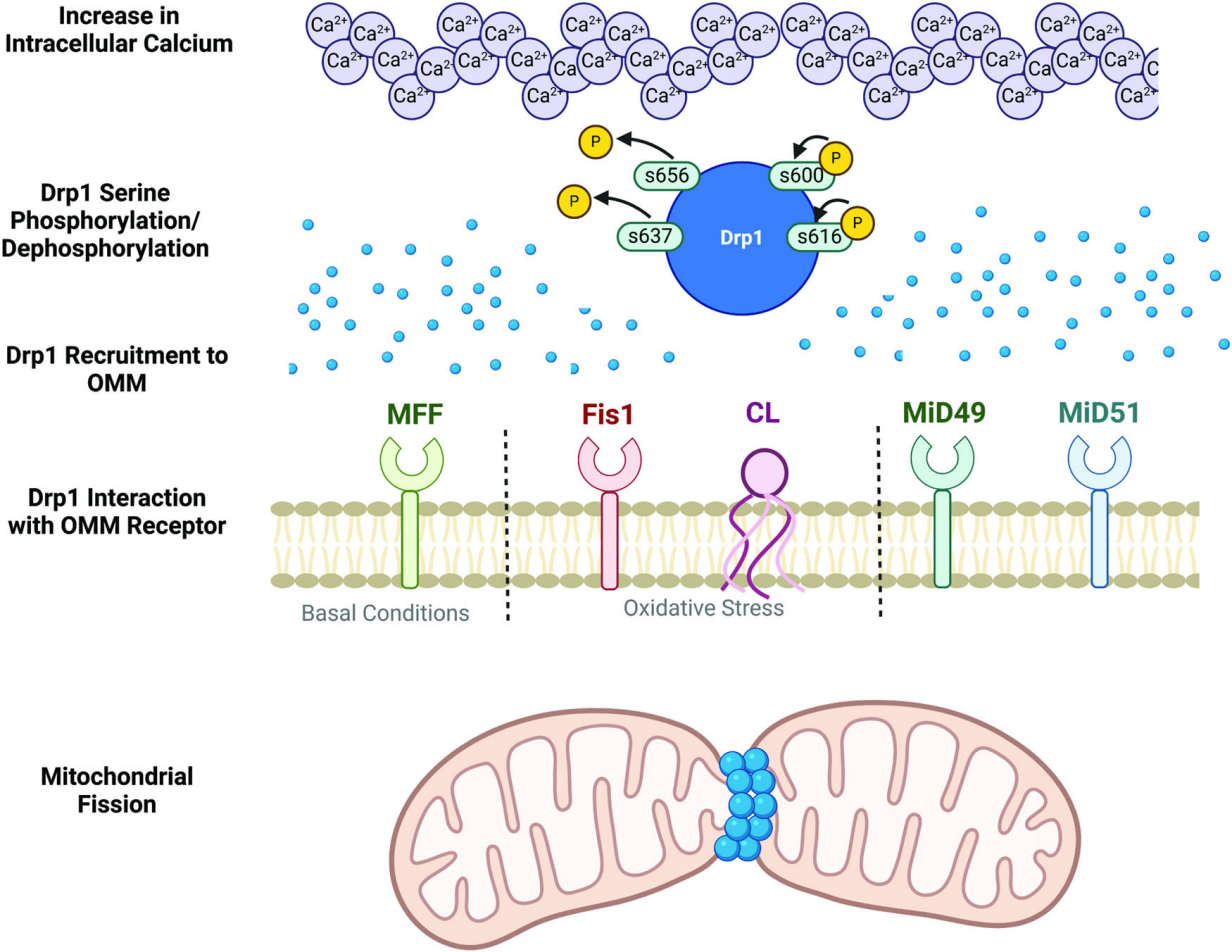
Mitochondrial fission is initiated by an increase in cytosolic calcium, which phosphorylates serine sites s600 and s616 and dephosphorylates serine sites s565 and s637 on Drp1 leading to its accumulation on the outer mitochondrial membrane (OMM). Once translocated to the OMM, Drp1 can interact with several OMM receptors that are known to initiate mitophagy such as mitochondrial fission factor (Mff), mitochondrial fission protein 1 (Fis1) and mitochondrial dynamic proteins (MiD49/MiD51). Externalized cardiolipin (CL) has recently been shown to interact with and stabilize translocated Drp1.

Mitochondrial fission is executed by the GTPase dynamin-related protein (Drp1) and can be driven by calcium transients, ROS, electron transport chain integrity, and mitochondrial membrane potential, although it is experimentally difficult to determine the singular causality of each of these variables [37–40]. Normally residing in the cytosol, Drp1 translocates to the OMM upon activation by post-translational modifications and an increase in cytosolic Ca^2+^ [41, 42], thereby regulating mitochondrial fission (Fig. 3). In hippocampal neurons, an increase in calcium influx via voltage gated dependent calcium channels resulted in an increase in mitochondrial fission via phosphorylation of Drp1 at s600 by CamK1alpha [43]. Further, phosphorylation of Drp1 at s656 by cyclic AMP dependent protein kinase A resulting in mitochondrial elongation is driven by increased calcium levels while dephosphorylation of s656 results in mitochondrial fragmentation [41].

Upon recruitment to the OMM, Drp1 interacts with four major receptors: Fis1, Mff, MiD49, MiD51, and MCL-1 [44–46]. While interactions between Drp1 and all four receptors are capable of executing fission, Mff (basal/physiological fission) and Fis1 (stress-induced fission) are the best characterized receptors (Fig. 3) [44, 47, 48]. In mouse embryonic fibroblasts, Mff gain and loss of function experiments resulted in significant activation or reduction of mitochondrial fission, respectively [44, 49]. Through the N-terminus, Mff binds Drp1 and promotes the formation of Drp1 oligomers and promotes mitochondrial fission [50]. In vivo and in vitro studies overexpressed saturated and unsaturated fatty acids individually and observed an increase in mitochondrial fission in the unsaturated fatty acid condition and increase in mitochondrial elongation in the saturated fatty acid condition [51]. This suggests that Drp1 is interacting directly with the headgroups of unsaturated phospholipids to induce the hydrolysis and scission of the mitochondrial membrane. In yeast, the Drp1 homolog, Dnm1, was observed to promote mitochondrial fission through self-assembly and GTP hydrolysis [52]. Cryo-EM of purified Drp1 revealed structural changes after Drp1 GTP hydrolysis, which leads to Mid49 dissociation and the shortening and curling of Drp1 oligomers promoting scission of the membrane [53]. Taken together, under basal conditions, Drp1 interacts with unsaturated membrane phospholipids and Mff to promote membrane scission via GTP hydrolysis across several experimental models.

Mitochondrial fission 1 protein (Fis1) has been implicated in stress-responsive mitochondrial fission. Fis1 is embedded in the OMM where its N-terminal 1a helix protrudes into the cytosol to bind cytosolic Drp1. When the 1a helix is mutated, Drp1 interactions are destabilized and fission cannot proceed, impacting mitochondrial morphology and overall bioenergetics [54]. The phosphorylation of Fis1 is critical for mitochondrial fission following oxidative stress [55]. In vivo and in vitro studies pharmacologically inhibiting the phosphorylation of Fis1 result in elongated mitochondrial morphology and improved cellular respiration following I/R injury [56].

Myeloid cell leukemia factor-1 (MCL-1) is also thought to promote mitochondrial fission through its interactions with Drp1. MCL-1 can be sliced into long and short isoforms (MCL-1S and MCL-1L, respectively). In vitro studies demonstrated that an increase in MCL-1S:MCL-1L contributes to hyperpolarization of the mitochondrial membrane potential and accumulation of Ca^2+^contributing to altered mitochondrial morphology and impact on the functionality of the mitochondrial network [57]. Autophagy was also observed to be increased in cortical neurons in MCL1 knockout mice, suggesting that MCL1 plays a critical role in mitochondrial quality control through its involvement in Drp1 mediated fission [58].

CL is also proposed to have a role in promoting Drp1 mediated fission. NMR experiments identified CL’s binding motif to be conserved with Drp1, suggesting a role in mitochondrial dynamics and a codependent relationship with Drp1 to promote network fragmentation following oxidative stress (Fig. 3) [59]. Cryo-EM studies further support the role of CL in Drp1-dependent fission, demonstrating that CL binding alters the Drp1 helical structure promoting GTPase activity and oligomerization [60]. Fluorescence spectroscopy studies were able to further elucidate the mechanism by which CL-Drp1 dependent fission occurs [61]. With high spatial density in the target lipid bilayer, recruited Drp1 is stabilized by CL localization, specifically the unconstrained, non-raft-associated, fluid-phase CL. These associations prime the membrane for remodeling where GTP hydrolysis is stimulated, which further induces CL rearrangement. The tendency of CL to undergo the transformation from a lamellar, bilayer arrangement to a hexagonal, non-bilayer arrangement while Drp1 and GTP are present, prime localized regions of the membrane for fission [61]. Oxidative stress promotes CL externalization to the OMM, suggesting that this externalization promotes Drp1 stability and can induce fission, revealing yet another vital role that CL has in maintaining a healthy mitochondrial network.

#### Mitochondrial fusion

**Fig. 4:**
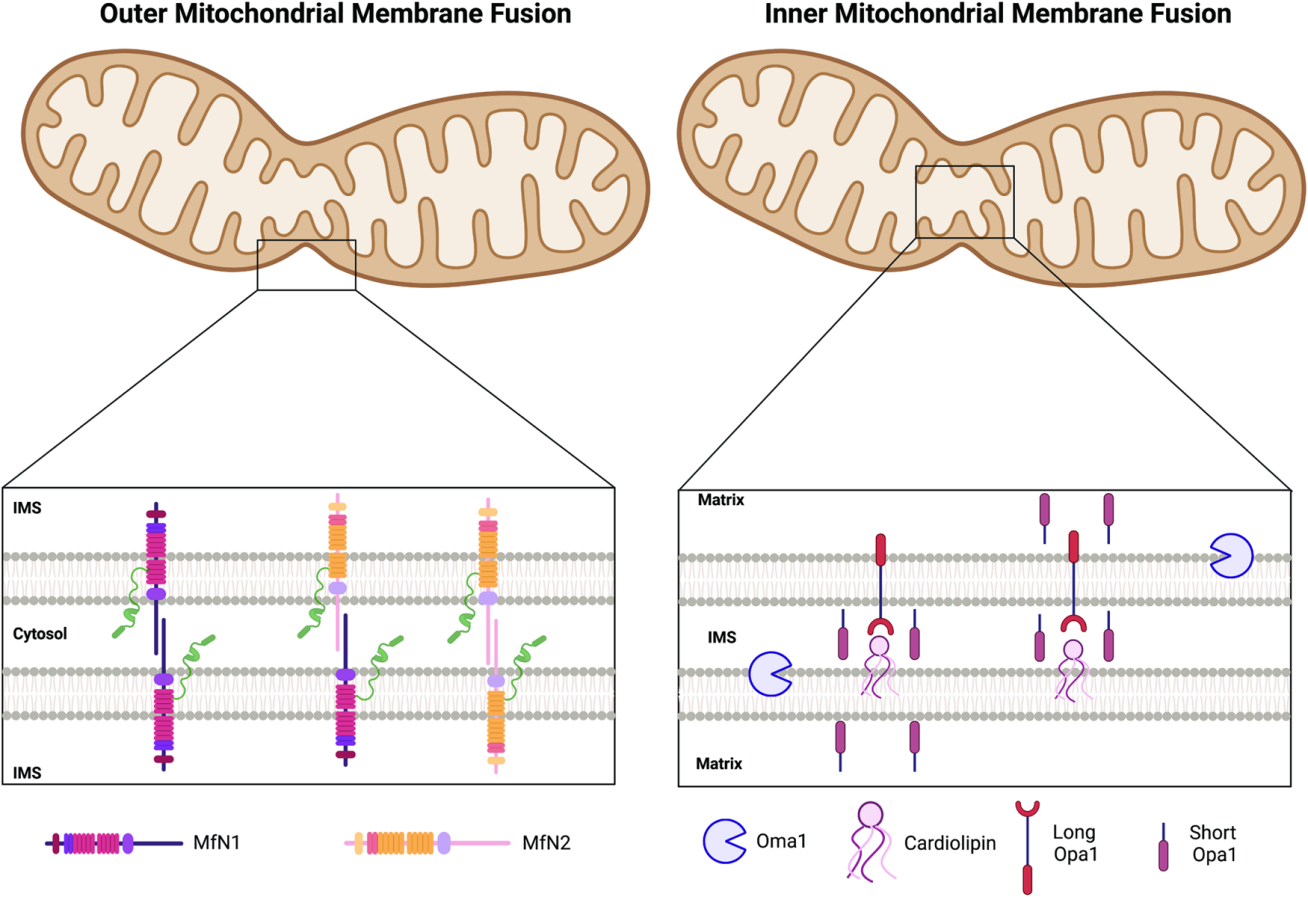
Mitochondrial fusion of the outer membrane is driven by mitofusin 1 and 2 (MfN 1/2), while the inner membrane requires a healthy balance of optic atrophy 1 (Opa1) isoforms. Cleaved by Oma1, Opa1 produces short and long isoforms, both of which support fusion. Membrane bound long - Opa1 interacts with cardiolipin on the opposing membrane in a GTPase dependent manner and promotes lipid fusion of the inner membrane.

Mitochondria are double membrane organelles and fusion requires merging of both the inner and outer membranes through distinct machineries. The key players in fusion of the OMM are the GTPases mitofusins 1 and 2 (Mfn1 and Mfn2) [62]. Upon GTP binding, Mfn1/2 unfold and adopt a stalk-like structure where they can form homo-oligomers or hetero-oligomers (Mfn1-Mfn1/Mfn1-Mfn-2/Mfn2-Mfn2) between the two adjacent mitochondria allowing fusion to occur (Fig. 4) [62–64]. In mice, knockouts of either of Mfn1/2 are lethal, but in vitro cell cultures are viable and result in a highly fragmented mitochondrial network [62, 64].

Similar to fission, mitochondrial fusion has bidirectional relationships with changes to the mitochondrial environment, including ROS, Ca^2+^, and bioenergetics [37]. Oxidized glutathione has been shown to drive fusion in HeLa cells and mouse embryonic fibroblasts [65]. Oxidized glutathione accumulation generates disulfide-mediated Mfn oligomers, thus priming the machinery required for fusion following exposure to cellular stress [66]. In vivo and in vitro studies found an increase in fusion following starvation, likely a response to limit oxidative stress to the mitochondrial network [67]. Consistent with fission, cytosolic Ca^2+^ levels have recently been suggested to play a role in modulating mitochondrial fusion [68]. This suggests that Ca^2+^ could affect mitochondrial homeostasis by promoting fission through stabilization of Drp1, while simultaneously impairing fusion.

At the IMM, optic atrophy 1 (Opa1) mediates both inner membrane fusion and cristae dynamics. Opa1-mediated membrane fusion requires a balance of short (S-Opa1) and long (L-Opa1) Opa1 isoforms [69, 70]. In HeLa cells, there are eight isoforms that are post-translationally cleaved, where long and short isoforms interact to regulate mitochondrial dynamics and morphology [70]. Long isoforms (L-Opa1) contain an N-terminal transmembrane anchor, whereas short isoforms (S-Opa1) do not [71, 72]. In vitro reconstitution of L-Opa1 and S-Opa1 individually and concurrently uncovered that both are necessary for proper fusion of the IMM [71], a result that has been replicated in yeast and cell culture [72, 73]. L-Opa is thought to contribute more to fusion while S-Opa contributes more to cristae support and mitochondrial energetics [71, 74]. Overexpression of S-Opa1 in an in vitro showed inhibition of fusion, whereas L-Opa1 alone was capable of membrane docking and content release [71]. Following oxidative stress, the mitochondria is depolarized resulting in Oma1 dependent cleavage of Opa1 (Figs. 4) [75].

During fusion, Opa1 oligomers create membrane protrusions with limited stability on adjacent OMMs. Mitochondrial contact site and cristae organizing system drive the structural organization and priming of the IMM and cristae for fusion [76, 77]. Lipid mixing between the two protruding membranes forms a fusion pore that can progress to complete fusion by Opa1 GTPase activity [78]. Interestingly, fusion of the IMM is CL dependent. The intermembrane space domain of L-Opa1 binds CL on the opposing membrane, forming a heterotypic dimer and in a GTP independent manner while priming fusion of the inner membranes (Fig. 4) [78]. L-Opa1-CL fusion occurs in a GTP dependent manner, with double the liposomes observed in the presence of GFP [71]. Purified human L-Opa1 was sufficient to interact with CL on opposing membranes and successfully fuse mitochondrial membranes in vitro, whereas purified human S-Opa1 was not sufficient to induce membrane fusion [79]. These studies support the necessity of L-Opa1 and CL interactions for proper membrane fusion through formation of heterotypic dimers on the opposing inner membranes and that S-Opa1 acts as a supporting role during tethering.

### Mitochondrial dynamics in I/R

Mitochondrial dynamics are known to be highly active during cerebral I/R injury and can modulate injury progression and severity. Opa1 processing appears to be a central component of mitochondrial fragmentation following I/R injury. HT22 cells exposed to oxygen glucose deprivation and reoxygenation (OGD/R) and rats exposed to global ischemia and reperfusion displayed an increase in Opa1 processing as well as cytochrome c release to the cytosol, activation of caspase 3/7 and initiated programmed cell death [80]. Similarly, following OGD/R in primary neurons and middle cerebral artery occlusion (MCAO) in mice, an increase in Opa1 cleavage was observed resulting in an accumulation of S-Opa1 contributing to excessive fragmentation of the mitochondrial network [81]. Accumulation of succinate, due to the reversal of succinate dehydrogenase following I/R injury, has been shown to drive fragmentation of the mitochondrial network [82]. This initial wave of fragmentation is generally attributed to ROS production at the onset of ischemia and broad mitochondrial depolarization throughout the ischemic phase [83]. The onset of reperfusion is thought to generate a large amount of ROS during the initial moments of reoxygenation [84]. Despite this ROS burst, mitochondrial fission slows or halts during early reperfusion, with variable onset of mitochondrial fusion [80, 84–86]. Intriguingly, fusion slows during the later stages of reperfusion, bringing forth a secondary wave of fission [80, 84, 86]. Primary neurons isolated from mt-Keima (a fluorescent mitophagy reporter) mice exposed to OGD/R showed a primary wave of fission at 6 h reperfusion and a secondary wave at 24 h reperfusion [86]. A similar result was found in a mouse model of neonatal hypoxic ischemic encephalopathy, where there was a primary wave of fission and fragmentation following the hypoxic ischemic insult (3 and 8 h reperfusion) and a secondary wave at 7 days [86]. Although the time scales differ with respect to model and severity of injury, it is clear that an initial wave of mitochondrial fragmentation occurs proximal to injury followed by a delayed, secondary wave. Upregulation in fission results in a fragmented mitochondrial network and an overall decrease in cellular energy reserves, commonly contributing to programmed cell death. A proposed hypothesis is that the initial fragmentation is a pathological response to the injury, followed by bioenergetic failure, high ROS production and mitochondrial permeability transition, which leads to a delayed secondary wave of fragmentation [80, 86–88]. Critically, modulation of mitochondrial dynamics can improve outcomes after neuronal I/R injury. Many studies targeted the initial wave of fission in the early stages of reperfusion by modulating Drp1 activity. Genetically ablation of Drp1 activating subunit (Bβ2) mitigated excessive fission and increased mitochondrial elongation which had an overall neuroprotective effect through increased respiratory capacity, stabilized Ca^2+^ levels, and reduced ROS production following I/R injury [89]. Pharmacological inhibition of Drp1 resulted in restoration of mitochondrial membrane potential following OGD/R in HT22 cells and a reduction in cerebral infarct in mice exposed to transient focal ischemia [89–93]. Additional studies focused on the role of fusion and expression of Mfn2 during reperfusion. Under normal and hypoxic conditions, Mfn2 depletion lead to an increase in programmed cell death, but overexpression of Mfn2 in hypoxic conditions increases cellular viability and provides neuroprotection through increased autophagosome formation and promotion of fusion [89–93]. Consistently, overexpression of L-Opa1 rescued mitochondrial morphology and improved overall cell viability in primary neuronal cultures exposed to I/R injury [89–93].

### Mitochondrial quality control

Maintenance of mitochondrial homeostasis is mediated by mitophagy which remodel and adapt mitochondria and their essential components. Defects in these mitochondrial quality control pathways are linked to neurological diseases and injuries.

#### PINK1/Parkin mitophagy

**Fig. 5:**
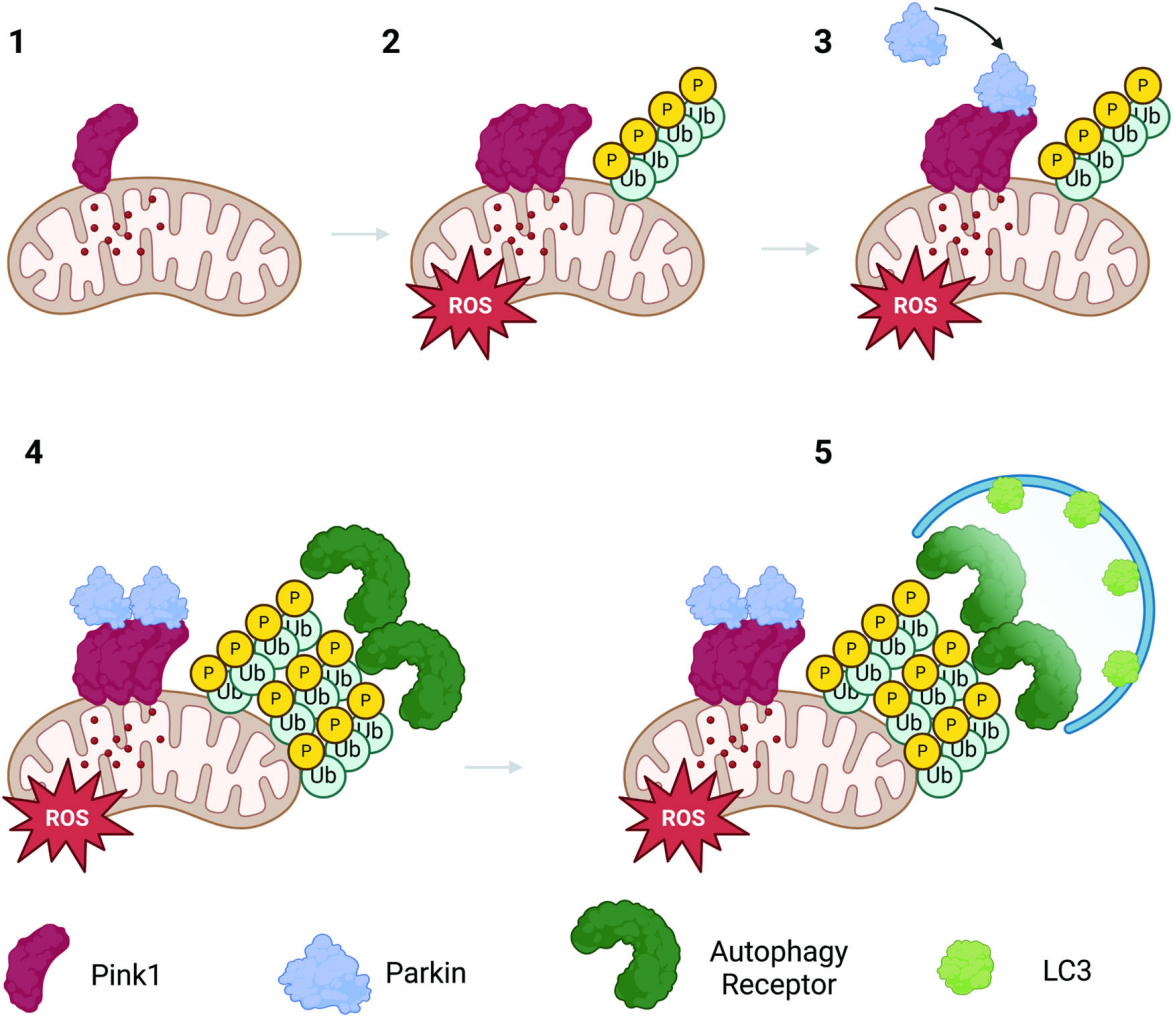
PTEN-induced kinase 1 (PINK1) is continually imported to the mitochondria where it is quickly degraded by intramitochondrial proteases. Import of PINK1 is dependent on mitochondrial membrane potential, when the membrane is depolarized the import of PINK1 comes to a halt, leading to its accumulation on the OMM. PINK1 then phosphorylates ubiquitin monomers and chains to recruit Parkin, an E3 ubiquitin ligase which is also phosphorylated by PINK1, generating more ubiquitin chains on the OMM. The phosphor-ubiquitin chains recruit autophagy receptors (e.g., OPTN) to mitochondria and eventually bind to microtubule associated protein light chain 3 (LC3) on photophores to initiate macro-autophagy.

Mitophagy, the selective autophagic degradation of mitochondria, includes engulfment of whole mitochondria as well as selective removal of mitochondrial components via mitochondrial derived vesicles [94, 95]. The best characterized form of mitophagy in neurons is the stress-responsive PINK1-Parkin pathway (Fig. 5). The kinase PINK1 is continually imported into mitochondria under basal conditions where it is quickly degraded by intramitochondrial proteases PARL and LonP1 [96, 97]. When mitochondria become depolarized, PINK1 import is halted, leading to accumulation of PINK1 on the OMM [96, 98]. At the mitochondrial surface, PINK1 phosphorylates nascent monomers and ubiquitin chains on OMM proteins, recruiting Parkin, a cytosolic-residing E3 ubiquitin ligase [99–101]. Similar to ubiquitin, PINK1 phosphorylates Parkin at S65 on its ubiquitin-like-domain to activate Parkin activity [102–104]. Parkin activation leads to ubiquitination of key OMM proteins, such as VDAC, hexokinase I, Tomm20 and RHOT1/2, generating more polyubiquitinated substrates for PINK1 kinase activity [100, 105–107]. Phosphorylation of Parkin-constructed ubiquitin chains stabilizes Parkin translocation and creates a feedforward loop between PINK1 and Parkin [102]. Although the specific linkage type of polyubiquitin chains on substrates determines the fate of the substrate, the majority are associated with initiation of macro-autophagy [108, 109]. K63-linked ubiquitin chains and ubiquitin monomers phosphorylated by PINK1 at S65 recruit the autophagy adapters Optineurin (OPTN), p62, and NDP52 to damaged mitochondria [99, 110–114]. Autophagy receptors then recruit LC3-expressing phagophores to mitochondria for engulfment, with conditional necessity of phosphorylation by the kinase TBK1 [113, 115]. Once the mitochondria is completely sequestered by the phagophore, it matures into an autophagosome that will eventually fuse with the lysosome for breakdown of the mitochondrial components.

#### Cardiolipin mitophagy

**Fig. 6:**
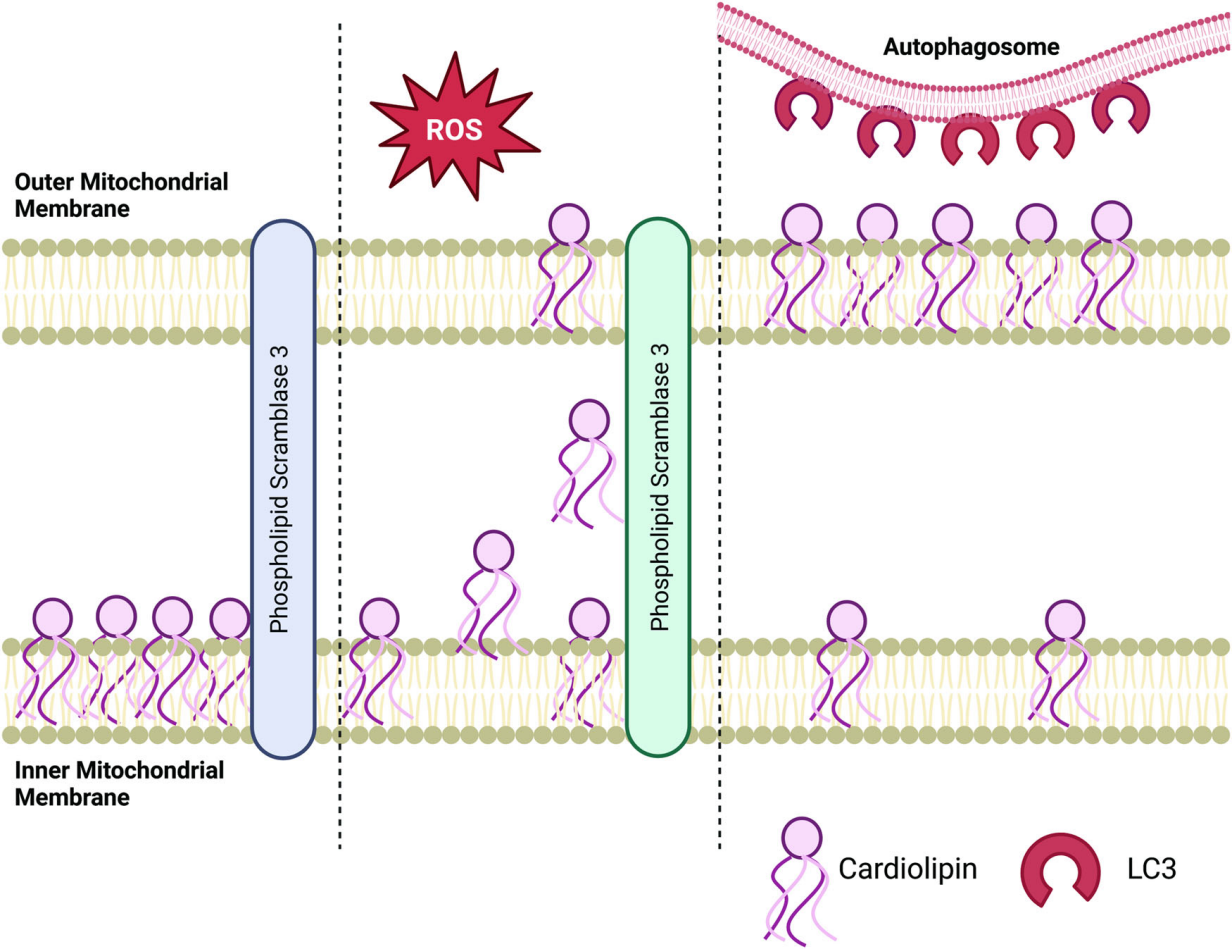
Cardiolipin driven mitophagy in response to oxidative stress. Cardiolipin typically found on the inner mitochondrial membrane is translocated by phospho scramblase 3 (PLSCR3) to the outer membrane following oxidative stress or reactive oxygen species (ROS) accumulation. Once externalized, cardiolipin is recognized by microtubule associated protein light chain 3 (LC3) on autophagosomes, initiating macro-autophagy.

The PINK1/Parkin pathway occurs under basal conditions, as general housekeeping to maintain a healthy mitochondrial network, however following injury, CL externalization is thought to induce mitophagy. When there is a decrease or partial collapse of the membrane potential, CL is externalized to the OMM where it contributes to CL-dependent mitophagy [25]. When CL is unbound from the IMM it is translocated to the OMM by phospholipid scramblase-3 (PLS3) where it is externalized and recognized by LC3 on the autophagosome (Fig. 6) [35, 116, 117]. Until fusion with the lysosome, membrane-bound lipidated LC-3II will remain attached to the autophagosome. Interestingly, LC-II has binding affinity with externalized CL [35]. The rate of mitophagy decreases in HeLa cells, SH-SY5Y cells, and in primary culture neurons when PLS3 is inhibited, preventing the translocation of CL to the OMM and supporting the role of CL in mitophagy initiation [35, 116, 117]. Another mechanism in which CL can contribute to mitophagy is through Beclin 1. In mitochondria, externalized CL has been shown to interact with Beclin 1, resulting in an induction of mitophagy [118]. Both of these interactions suggest the role of CL in inducing mitophagy in response to stress in various models.

### Mitochondrial quality control in I/R

The role of mitophagy in clearing damaged and dysfunctional mitochondria suggests it is an important component of recovery from I/R injury. In cell animal models of I/R injury, autophagy and mitophagy machinery are upregulated, along with mitophagic flux [68, 119–121]. Despite extensive study, the importance and timing of mitophagy induction remain unclear with reports of activation in the ischemic and reperfusion phases of injury [68, 119–121]. These studies focused on various timepoints of reperfusion ranging from immediately following reperfusion to 24 h after, all reporting significant upregulation in mitophagy [122–124].

While the occurrence of mitophagy following I/R injury is well-documented, the relationship between mitophagy and neurological outcomes after I/R injury is debated. Conflicting reports in similar models present mitophagy as either neuroprotective or an overactive mechanism potentiating mitochondrial dysfunction. Autophagy inhibition (pharmacological and genetic) during reperfusion exacerbates neuronal injury [121]. This suggests that autophagy can be protective during reperfusion, likely attributed to mitophagy related mitochondrial clearance. Similarly, PINK1 knockout exacerbated cell death and cerebral infarct volume in models of I/R injury, whereas overexpressing PINK1 had a rescue effect [125]. These results are supported by evidence that overexpressing PINK1 provides neuroprotection by preventing the release of cytochrome *c* and mitigating programmed cell death through improved mitochondrial function [126]. Under basal conditions PINK1/Parkin machinery are involved in the biogenesis of mitochondrial derived vesicles responsible for discarding dysfunctional mitochondrial proteins to maintain mitochondrial homeostasis and a functional network [95]. Following injury, there is an accumulation of oxidative stress that stimulates PINK1/Parkin mitophagy and possibly contributes to an increase in mitophagy and degradation of the mitochondrial network. Activation of mitochondrial degradation could lead to insufficient energy reserves and an overall decrease in cell health.

### Interplay between mitochondrial dynamics and QC pathways

Mitochondrial dynamics and quality control systems display coordinated activity to maintain mitochondrial homeostasis. Among the best characterized relationships is the isolation and clearance of dysfunctional mitochondria through fission and mitophagy [14]. In the context of cerebral injury, increased mitochondrial fission precedes the surge in mitophagy, although this relationship may be correlative as both fission and mitophagy are triggered by mitochondrial depolarization and dysfunction [127–130]. Evidence supports a causal role of fission and mitophagy in neuronal injury. The predominant paradigm suggests limiting fission and augmenting mitophagy is neuroprotective. Tang et al. demonstrated reduced mitochondrial damage and rescue of Neuro2a cells by Drp1 knockdown or Parkin overexpression prior to OGD/R, concluding that Parkin-mediated mitophagy can be protective through degradation of excessive Drp1 mediated fission [131]. Further, an upregulation in Parkin mediated mitophagy was observed in hippocampal neurons following transient global cerebral ischemia and improved neurological outcomes [132]. Differentiating mitophagy and autophagy, in vitro studies showed that moderate levels of ROS induced Drp1-dependent mitophagy but not non-selective autophagy [128]. Additional studies expanded on this, demonstrating that Fis1 can stimulate non-selective autophagy but only in response to mitochondrial function rather than stimulating fission and ultimately mitophagy [127–130]. Further, when mitochondria undergo fission, depending on where fission is occurring on the membrane, they produce two daughter mitochondria. One daughter mitochondria has higher membrane potential with higher probability of undergoing fusion and one has a lower membrane potential with a higher probability of undergoing mitophagy [127–130]. When taken together, these studies imply a strong connection between fission and mitophagy, isolation of damaged mitochondria followed by its degradation. Burman et al. proposed a more nuanced model in which Drp1-mediated mitochondrial fission acts a protector of healthy mitochondria by severing dysfunction segments, allowing for PINK1/Parkin mitophagy to proceed only on fission-produced dysfunctional mitochondria [133]. Aside from PINK1/Parkin mitophagy, Drp1 is required for the biogenesis of mitochondrial-derived vesicles, which act to selectively remove mitochondrial proteins and mitochondrial DNA-containing nucleoids [95, 134, 135]. As a part of the pseudo-ubiquitin cascade that occurs during PINK1/Parkin mitophagy, the outer membrane fusion protein Mfn2 becomes ubiquitinated. Ubiquitination of Mfn2 can lead to both selective degradation of Mfn1/2 complexes, as well as increased autophagy receptor recruitment for mitophagy initiation, thereby inhibiting fusion and enhancing mitophagy [108, 136]. Similarly, Mfn2 inhibition and knockout decreases general autophagy and mitophagy, and Mfn1 overexpression increases mitophagy, further strengthening this relationship [136–139]. These effects may be specific to Mfn1/2 rather than fusion, as overexpression of Opa1 leads to decreased mitochondrial sequestration in autophagosomes [130]. Many outstanding questions remain as to the relationships between mitochondrial fusion and mitochondrial quality control pathways that prompt further investigation.

Although CL dependent mitophagy is well established, the possibility that it contributes to other forms of mitophagy is well supported. In several in vitro models, increase in the expression of CL, which is thought to rescue the complex 1 deficits seen in the PINK1 mutants [140]. The mechanism by which this is occurring is not entirely known, but the increase in CL expression is thought to restore the electron transfer between complex 1 and ubiquinone [140]. In the context of acute lung injury, PINK1 was shown to bind to and degrade cardiolipin synthase 1 (CRLS1) and decrease overall CL expression resulting in dysfunctional mitochondria and increased programmed cell death [141]. These effects were restored in PINK1 knockout mice and overall acute lung injury was decreased. Although these interactions are in the realm of neurological disorder and mitochondrial injury, they suggest that CL regulates mitochondrial health on a grander scale than current literature suggests.

In addition to mitophagy, CL plays several roles in I/R injury and overall mitochondrial stress response. Due to its molecular and chemical structure, CL has a strong ability to form non-covalent interactions with several proteins involved in cellular respiration (complex I, II, IV, and V), carrier proteins (ADP-ATP carrier, phosphate carrier, uncoupling protein) and peripheral membrane proteins (cytochrome c and creatine kinase) [142]. In vitro I/R studies showed that exogenous oxidized CL lowered the Ca^2+^ threshold for opening the mitochondrial mPTP and promoting the release of cytochrome c from the mitochondria [143]. When Ca^2+^ levels increase, CL becomes oxidized which destabilizes its non-covalent interactions with a number of proteins outlined above, which can contribute to several mitochondrial stress responses, including opening of the mPTP. Further, CL content increases in response to mitochondrial stress which can stabilize mtDNA, a known DAMP which contributes to the immune response. ROS also triggers an increase in peroxisome proliferator-activated receptor gamma coactivator-1α (PGC1α) which promotes mitochondrial biogenesis as an attempt to restore energy production and reestablish a healthy mitochondrial network. Interestingly, recent studies have shown that CL synthesis is influenced by PGC1α [144]. As an integral phospholipid, CL plays several crucial roles not only to membrane integrity but also to maintaining healthy mitochondria, individually and as a network as a whole.

## Conclusions

Mitochondria are dynamic organelles that have complex physiology in order to maintain a healthy network and adequate cellular energy supply. Mitochondrial dynamics and quality control are an integral part of maintaining mitochondrial network homeostasis. While basal levels of fission and mitophagy maintain a healthy mitochondrial network, overactive fission can result in a fragmented, dysfunctional mitochondrial network. Mitochondrial fusion is the primary mechanism of equilibration across the mitochondrial network of a cell, as few other mechanisms exist for distribution and dispersal of mitochondrial proteins, DNA, and metabolites. When either the outer or inner mitochondrial membrane cannot properly fuse, the result is a fragmented mitochondrial network with decreased cellular respiration and energy reserves. Further work is required to disentangle the beneficial and detrimental effects of mitophagy in I/R, however, much of the variability may be related to injury severity, neuronal cell types and model organisms.

Lipids are highly susceptible to the oxidative injury characterized in I/R injury. CL is an essential phospholipid found on the IMM that contributes to cristae structure, membrane curvature and cellular respiration. Due to the variety and tissue specificity of the four acyl chains and their sensitivity to oxidation, CL has also been used as a biomarker in several diseases and disorders. In the early pathology of Type II diabetes, oxidized CL has been observed in circulating plasma [145]. Altered CL profiles have been observed in Alzheimer’s and Parkinson’s Disease [146]. Neuronal specific CL has been found in circulating blood and used to predict injury severity in rodent models of pediatric traumatic brain injury and adult cardiac arrest [147, 148]. As identification techniques advance, the role of CL in injury and disease pathologies can be more accurately and reliably assessed. Advanced mass spectrometry methods would improve investigation into how the acyl chains respond to and are altered by oxidative stress which will allow researchers to better understand how membrane dynamics play a role in injury progression. With this information, better diagnostics, therapeutics, and pharmacological interventions could be designed. Research is just scratching the surface with CL as new findings are uncovering novel relationships between CL and known proteins involved in mitochondrial dynamics and quality control and the importance of these mechanisms to I/R injury.
